# Mental health among patients with chronic musculoskeletal pain and its relation to number of pain sites and pain intensity, a cross-sectional study among primary health care patients

**DOI:** 10.1186/s12891-022-06051-9

**Published:** 2022-12-22

**Authors:** Kirsti Krohn Garnæs, Siv Mørkved, Torgrim Tønne, Lars Furan, Ottar Vasseljen, Hege Hølmo Johannessen

**Affiliations:** 1 Department of Public Health and Nursing, Faculty of Medicine and Health Sciences, Norwegian University of Science and Technology (NTNU), P.O. Box 8905, N-7491 Trondheim, Norway; 2 Department of Obstetrics and Gynaecology, St. Olavs Hospital, Trondheim University Hospital, P.O Box 3250, Trondheim, Norway; 3 Central Norway Regional Health Authority, P.O Box 464, Stjørdal, Norway; 4 Tiller Physiotherapy and Manual Therapy. Ivar Lykkes Veg 9, 7075 Tiller, Norway; 5 Stokmoen Physiotherapy, Wergelandsveien 27, 7504 Stjørdal, Norway; 6 Department of Health and Welfare, Østfold University College, Kobberslagerstredet 5, Fredrikstad, Norway

**Keywords:** Chronic musculoskeletal pain, Mental health, Pain sites, Pain intensity, Burden of disease

## Abstract

**Background:**

Chronic musculoskeletal pain (CMP) is characterised by pain related to the muscles or the joints with a duration of three months or more and is associated with high symptomatic burden in patients in primary health care. CMP is commonly associated with impaired mental health, which may affect the rehabilitation process. The primary aim of this study was to compare symptoms of anxiety, depression, fatigue, and insomnia in patients in primary health care with and without CMP. The secondary aim was to assess difference in mental health symptoms related to number of pain sites and pain intensity.

**Methods:**

This cross-sectional study was conducted in Trondheim, Norway. All patients aged 21–58 from randomly selected general practitioners (GPs) were invited to participate. Participants were classified into two groups according to presence of CMP. Symptoms of anxiety, depression, fatigue, and insomnia were assessed by the Hospital Anxiety and Depression Scale (HADS), Chalder Fatigue Questionnaire (CFQ), and Insomnia Severity Index (ISI), respectively, using an online survey system.

**Results:**

From the patient lists of six GPs, we included 969 patients. Mean age 46 years (SD: 10.1), and 517 reported CMP. CMP patients reported higher mean symptom score for anxiety (5.4 vs 3.7), depression (3.4 vs 2.0), fatigue (14.2 vs 11.2), and insomnia (8.1 vs 4.4), all *p* < 0.01 compared to no-CMP patients. Symptoms of impaired mental health increased with increasing number of pain sites and pain intensity (*p* < 0.001).

**Conclusions:**

Primary health care patients with CMP reported significantly more symptoms of anxiety, depression, fatigue, and insomnia than patients without CMP. The higher number of pain sites and pain intensity, the more mental health symptoms, especially of anxiety. Primary health care personnel have to address mental health issues when treating patients with CMP.

**Trial registration:**

Clinicaltrials.gov (NCT02020772, 25/12/2013).

## Introduction

Musculoskeletal pain is the most prevalent cause of disability worldwide [1, 2], and represents the leading cause of seeking health care from general practitioners (GPs) in Norway [3]. Musculoskeletal pain can be single-sited, but is most often multi-sited [4]. When musculoskeletal pain persists for more than three months, with continuous or repeated pain episodes, it is defined as chronic [5]. Approximately 20–30% of the adult working population experience chronic musculoskeletal pain (CMP), and the numbers are rising [3, 6–8].

CMP in combination with reduced physical function is associated with increased risk of mental health issues, such as fatigue [9–11], sleeping problems [12–14], depression, and anxiety [15–21]. CMP therefore often represents multiple challenges for the individual’s daily functioning and quality of life [22–24]. There is evidence that prognostic factors as functional disability, widespread pain and previous pain episodes, are shared across patients with different CMP complaints [25, 26].

Non-musculoskeletal pain symptoms as reduced work capacity, and impaired social life represent independent predictors of quality of life among CMP patients [27, 28]. In long lasting musculoskeletal pain, mental health issues may affect the ability to cope with and recover from chronic pain [29–31]. Considering that CMP patients represent a major burden to the primary health care system, there is a need for a more comprehensive understanding of factors associated with musculoskeletal complaints and prolonged disability, as mental health. To develop and improve treatment strategies at a societal and systemic level, targeting the CMP patient group as a hole and addressing mental health status is very important.

We have previously published findings on reduced health-related quality of life in a CMP group compared to a no-CMP group in a group of unspecified patients in the primary health care system [32]. In this paper we aimed to investigate the prevalence of symptoms related to mental health, such as anxiety, depression, fatigue, and insomnia among patients with CMP compared to patients without. In addition, we have among the CMP patients investigated mental health symptoms related to perceived pain intensity and number of pain sites.

## Methods

### Study design

This is a cross-sectional study based on data collected from patients of GPs in Trondheim County, Norway. The data was collected in a randomized controlled trial (RCT) conducted in the period from November 2013 to July 2015. The RCT aimed to investigate patients in primary health care clinical practice; identify CMP, and to compare the effect of systemized collaboration between GP’s and physiotherapist on sick leave and referral to special health care. The focus in the present study is to compare mental health symptoms among patients in primary health care with and without CMP. The study was performed at the Department of Public Health and Nursing, Faculty of Medicine and Health Sciences, Norwegian University of Science and Technology (NTNU), Trondheim, Norway.

### Subjects

About 211 106 citizens lives in Trondheim municipality (2022), which is the fourth largest city in Norway. Among 162 general practitioners in Trondheim municipality, we randomly invited 20 to participate in the study. The 20 GPs were randomly selected using a computer random number generator, developed and administrated independently by the Unit for Applied Clinical Research, Norwegian University of Science and Technology (NTNU), Trondheim, Norway. GPs were stratified by gender before the randomization to prevent possible bias related to differences in the GPs’ patient populations. We used postal mail and telephone to invite GPs to participate in our study. To ensure rural GP representation, two additional GPs (one male and one female) were selected due to experience with interdisciplinary collaboration in primary health care, invited, and accepted our invitation to participate in the study.

We invited all patients registered on the participating GPs’ patient lists aged 21 to 58 years to participate in the study. In the original study, the main aim was to explore sick-leave in a working population during a three-year period from inclusion. In Norway, most adults younger than 21 are in full-time education and many have the opportunity of early retirement at the age of 62 years. Thus, aiming for enrolment of a population most likely to be working throughout the study period, the age range 21 to 58 years was chosen. Except from this, our study included no exclusion criteria. Eligible patients received study information and invitation to participate by postal mail. The patients accepted the invitation by replying to the invitational letter in writing, by phone, or by completing the web-based questionnaire by logging in to the online survey system (CheckWare®, Norway).

### Measures

The data in this study was collected by self-reported questionnaires using an online system (CheckWare®) sent to the participants registered private email account. The participants had to complete the questionnaires within one month, if not, an automatic reminder was sent. Patients were classified with CMP if reporting “pain and/or stiffness related to the musculoskeletal system, with continuous duration of at least 3 months during the last year”.

Subject characteristics, including gender, age, marital status, education, occupational activity, sick leave, grade of social benefits, and bodily pain were self-reported by study participants at study entry using the online survey system. Questions regarding pain sites and pain intensity were included in the same questionnaire, but these questions were only available for the subjects reporting CMP. The questions regarding pain sites and pain intensity were highlighted to relate to the episode(s) of CMP, not necessary to the time point of answering the questionnaire. Pain characteristics used in the current study were assessed by questions based on the “SF-36 Health Survey (SF-36®/SF-36v2®, Ware JE; Sherbourne CD, 1990,1998)” [33, 34], the “Nordic musculoskeletal questionnaire” (NMQ) [35], and the “Brief Pain Inventory Long—Form (BPI)” [36]: The patients registered location and number of pain sites in a digital body map alongside the questionnaire. The pre-specified response alternatives for pain sites were; head, neck/shoulder, shoulder, upper back, chest, elbow, abdomen, lower back, hand, hip/thighs, knee, ankle/foot. In the statistical analyses we grouped the pain areas into six; head/neck/shoulder, chest/abdomen, elbow/hand, upper back, lower back, hip/thigh/knee/ankle/foot. We assessed pain intensity by asking the patients: “Please rate the intensity of current perceived pain (range 0 to 10)”, and pain was rated using an 11-point numerical rating scale ranging from “0” (No pain), to “10” (Worst pain imaginable). More detailed description of the pain assessments in this study are previously published [32]. Before analysing the data, pain intensity was categorized into none to very mild pain (0–2 points) mild pain (3–5 points) moderate pain (5–7 points), and severe/very severe pain (8–10 points). For the statistical analyses, number of pain sites was reported on a scale from 1 to 12, and categorized into 0–2, 3–5, 6–8, 9–12 pain sites.

We assessed symptoms of anxiety and depression, referring to the last week, by the Hospital Anxiety and Depression Scale (HADS) [37]. HADS is a frequent used questionnaire in primary care and in research, well validated and reliable tested, aiming to investigate symptoms of anxiety and depression [38, 39]. The questionnaire contains 14 questions, scored from 0 to 3 points, where higher scores indicate more symptoms of anxiety and/or depression. The 14 questions are separated into two sub-scales, one for anxiety and one for depression. Both the anxiety and depression subscale total scores range from 0 to 21 points, and a score below 7 points represents no signs of anxiety or depression, from 8 to 10 represents borderline cases, and above 11 indicates clinical anxiety or depression [37].

We assessed fatigue by the Chalder Fatigue Questionnaire (CFQ) [40]. The CFQ is a commonly used and well validated questionnaire in general populations and often used among patients in a primary health care settings [41]. It has previously been validated in a Norwegian population [42]. The questionnaire measures the severity of fatigue experienced during the last month on a four-point Likert scale; “less than usual”; “same as usual”; “more than usual”; and “much more than usual”. The 11-item fatigue scale measures both physical and mental features of fatigue. The global score ranges from 0 to 33 points, physical fatigue sub-score from 0 to 21 points, mental fatigue sub-score from 0 to 12 points, and higher scores indicate greater fatigue.

Sleep disturbances were assessed by the questionnaire Insomnia Severity Index (ISI) [43]. This questionnaire is a reliable and valid measure often used in insomnia research [39, 44]. ISI contains 7 items, scored from 0 to 4 points, with higher scores indicating higher insomnia severity. The total score ranges from 0 to 28 points. A score between 0 to 7 points represents no clinically significant insomnia, 8 to 14 points represent sub-threshold insomnia, 15 to 21 points represent moderate clinical insomnia, and 22 to 28 points represents severe clinical insomnia [43].

### Ethics

This study was approved by The Regional Committee for Medical and Health Research Ethics in Central Norway (2012/1232), and our study procedures followed the Helsinki Declaration. All participants completed an informed consent before they entered the study and the online questionnaires. All participants who participated in our study were part of a prize draw for a gift card worth 500 NOK.

### Statistics

Patient characteristics, comparisons of self-reported symptoms of anxiety, depression, fatigue and insomnia between patients with and without CMP were analysed by independent samples t-test for continuous variables, Fisher’s Exact tests for categorical variables, Pearsons Chi-square test for dichotomous variables, and Mann–Whitney U-test for ordinal variables. Normality of the continuous data was assessed by Shapiro–Wilk. The results (observed data) are presented as number (n) and percentages (%) or means and standard deviation (SD) as appropriate. Differences between the groups are presented in mean difference and 95% confidence interval (CI) and *p*-value.

Differences in symptoms between the various sub-groups of pain sites and pain intensity were assessed using a Kruskal–Wallis one way ANOVA, adjusted for the Bonferroni correction for multiple tests. Due to outliers, we log transformed the data by using the function Log10 before the ANOVA analyses were performed. Missing data was handled by listwise deletion, thus only observed data were included in our analyses.

The current study is based on an RCT, thus no sample size calculation was performed for the thesis included in this paper. We considered *p*-values < 0.05 as statistically significant. The statistical analyses were conducted using IBM SPSS Statistics version 27.

## Results

**Fig. 1:**
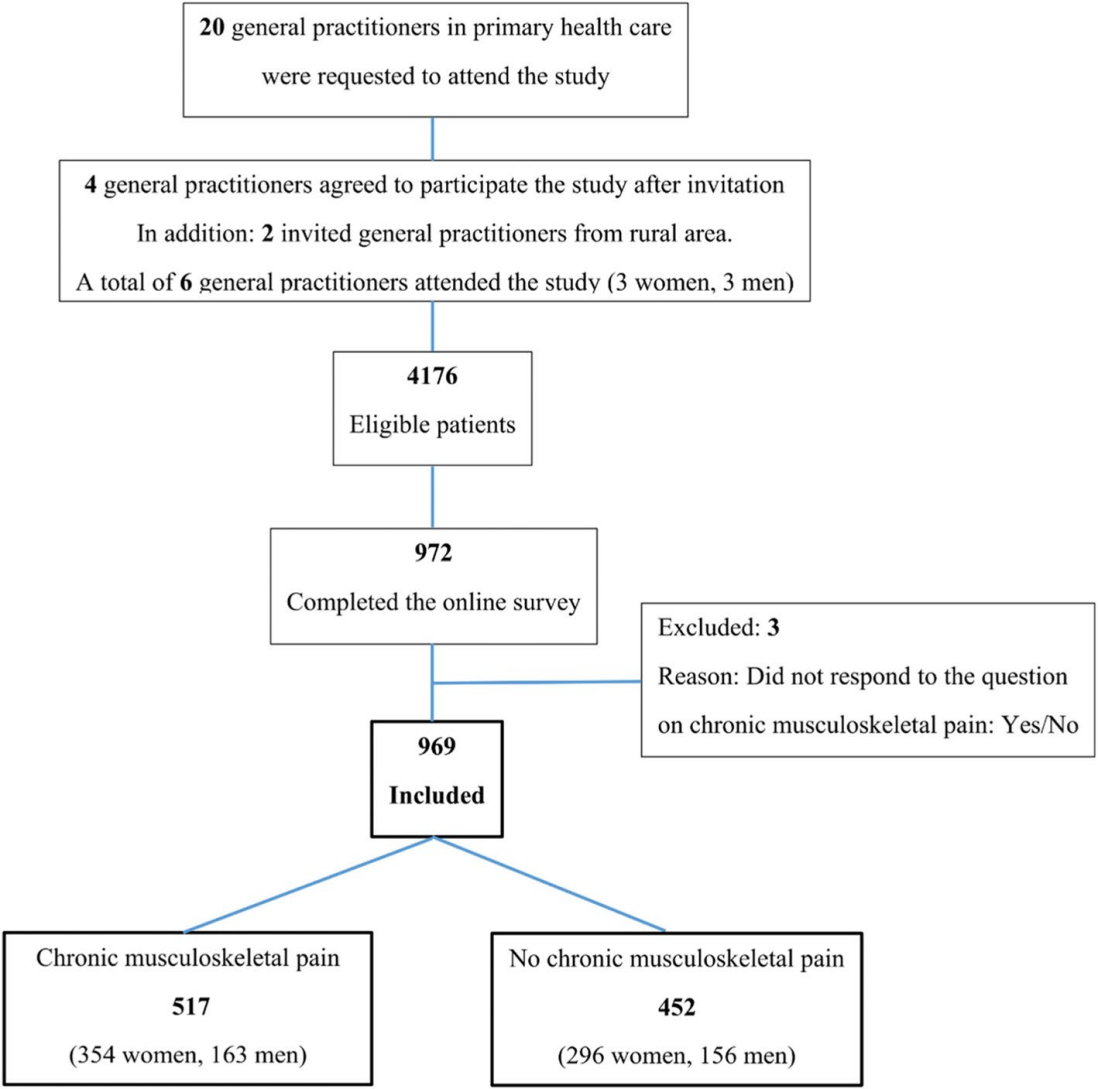
Flow chart of the study population, patients in primary health care with and without self-reported chronic musculoskeletal pain (*n* = 969)

In total, six GPs agreed to attend our study. Among the twenty GPs invited to the study, four agreed to participate (two female and two male), in addition to the two invited GPs from rural area (one female and one male). Study flow chart is presented in Fig. 1. Further detailed description of recruitment, and pain characteristics for the patients with CMP is previously published [32].

Patient characteristics is presented in Table 1, these data are previously published. Twice as more women than man participated in our study. About one third of the participants were in the age group 41–50 years. The patients who reported CMP differed somewhat from the patients without CMP, by being slightly older, less educated, and a higher amount working part-time. We found an overall low rate of seek leave in both groups, however the CMP-group represented a statistically significant higher rate compared to the group reporting no CMP. Among the patients with CMP statistically significant more patients reported a bodily pain duration of more than 6 months, compared to the no-CMP group. In addition, the CMP patients experiencing more severe unspecified body pain compared to the no-CMP group. Among the patients with CMP, moderate pain intensity was most prevalent, mean number of pain sites were 3.5 (SD 2.3), and the lower back was most frequent reported pain site. These characteristically data are also previously published [32].

**Table 1:** Demographic characteristics of participating patients, grouped by those with and without self-reported chronic musculoskeletal pain (CMP), (*N* = 969)

***Subject characteristics***	***Patients with CMP (n* = *517)***	***Patients without CMP (n* = *452)***	
	*n* (%)	*n* (%)	*P-*value
***Gender***			0.36
*Female*	354 (68.5)	296 (65.7)	
*Male*	163 (31.5)	156 (34.3)	
***Age (years)**** mean (SD)*	45.6 ± 10.1	42.6 ± 10.2	˂0.01
*21–30 years*	57 (11.3)	66 (17.8)	
*31–40 years*	88 (17.5)	101 (23.3)	
*41–50 years*	156 (31.0)	134 (30.9)	
≥ *51 years*	202 (40.2)	121 (27.9)	
*Missing*	*14 (2.7)*	*17 (3.8)*	
***Marital status***			0.13
*Married/cohabitant*	405 (78.6)	348 (77.5)	
*Single*	74 (14.4)	77 (17.1)	
*Divorced/Separated/Widowed*	36 (7.0)	24 (5.2)	
*Missing*	*2 (0.4)*	*3 (0.7)*	
***Education***			˂0.01
*Primary/secondary school*	175 (34.0)	106 (23.5)	
*University ˂ 4 years*	141 (27.4)	137 (30.4)	
*University ˃ 4 years*	172 (33.4)	190 (42.1)	
*Student/other*	27 (5.2)	18 (4.0)	
*Missing*	*2 (0.4)*	*1 (0.2)*	
***Occupational activity***^***a***^			
*None*	28 (5.4)	15 (3.3)	0.12
*Full time work*	358 (69.2)	348 (77.0)	˂0.01
*Part time work*	80 (15.5)	57 (12.6)	0.23
*Under education*	33 (6.4)	31 (6.9)	0.80
*Disability pension*	35 (6.8)	3 (0.7)	˂0.01
***Receiving any form of benefit payments***	369 (71.4)	309 (68.4)	0.33
***Current 100% sick leave***	31 (6.4)	10 (2.3)	˂0.01
*Missing*	*31(6.0)*	*10 (1.8)*	
***Bodily pain duration* ≥ *6 months***	420 (81.6)	58 (12.1)	˂0.01
*Missing*	*2 (0.4)*	*4 (0.9)*	
***Bodily pain intensity lasting* > *3 months***			˂0.01
*None/very mild*	23 (4.5)	213 (47.7)	
*Mild*	171 (29.5)	133 (29.8)	
*Moderate*	217 (42.1)	83 (18.6)	
*Severe/very severe*	105 (20.3)	18 (4.0)	
*Missing*	*1 (0.2)*	*5 (1.1)*	
***Number of pain sites ***^***b***^			
*0–2*	208 (40.2)		
*3–5*	214 (41.4)		
*6–8*	73 (14.1)		
9–12	21 (4.1)		
***Pain sites***			
*Head/neck/shoulder*	87 (16.8)		
*Chest/abdomen*	176 (34.0)		
*Elbow/hand*	16 (3.1)		
*Upper back*	134 (25.9)		
*Lower back*	235 (45.5)		
*Hip/thigh/Knee/ankle/foot*	39 (7.5)		
***Current pain intensity***^b^			
*0–2 No to little pain*	238 (46.0)		
*3–5 Low to moderate*	195 (37.7)		
*6–8 Severe*	73 (14.1)		
*9–10 Very severe to worst pain ever experienced*	3 (0.6)		

Numbers are presents as *n* (%) unless otherwise stated

Statistics: Independent sample T-test for continuous variables, Fishers Exact Test for categorical variables, Pearsons Chi-squared test for dichotomous variables, and Mann–Whitney U-test for ordinal variables

*Abbreviations*: *CMP* Chronic musculoskeletal pain, *SD* Standard deviation

^a^Multiple response alternatives were allowed for occupational activity

^b^Only reported by CMP patients

### Mental health symptoms in patients with and without CMP

The total group of patients included in the current reported a overall good mental health. However, when comparing symptoms between the CMP patients and the no-CMP patients, the patients who reported CMP reported significantly more symptoms of anxiety, depression, fatigue and insomnia (Table 2), and more cases of symptoms score representing clinical anxiety and/or depression. The patients with CMP also reported significantly more symptoms of fatigue, especially physical fatigue.

**Table 2:** Symptoms of anxiety, depression, fatigue, and insomnia among patients with and without self-reported chronic musculoskeletal pain (CMP). Observed data in each group is presented as number and percent or mean and standard deviation (SD), comparison between groups with mean difference, 95% Confidence Interval (CI) and *p*-value

	**Patients with CMP** ***(n* = 517)**	**Patients without CMP** **(*n* = 452)**	**Comparison between groups**		
	*n (%)*	*n (%)*	*Diff*	*95% CI*	*p-value*
**Anxiety,** *mean* ± *SD*	5.4 ± 3.9	3.7 ± 3.0	-1.8	-2.2, -1.3	˂0.01
No anxiety	350 (72.0)	368 (88.7)			
Borderline anxiety	76 (15.6)	33 (8.0)			
Clinical anxiety	60 (12.3)	14 (3.4)			
*Missing*	*31*	*37*			
**Depression,** *mean* ± *SD*	3.4 ± 3.3	2.0 ± 2.5	-1.4	-1.8, -1.1	˂0.01
No depression	432 (88.7)	407 (95.3)			
Borderline depression	30 (6.2)	17 (4.0)			
Clinical depression	25 (5.1)	3 (0.7)			
*Missing*	*30 (5.8)*	*25 (5.5)*			
**Fatigue**					
Total score, *mean* ± *SD*	14.2 ± 4.9	11.2 ± 4.2	-3.0	-3.6, -2.4	˂0.01
*Missing*	*48 (9.3)*	*40 (8.8)*			
Physical fatigue sub-score, *mean* ± *SD*	9.7 ± 3.7	7.3 ± 3.1	-2.4	-2.8, -2.0	˂0.01
*Missing*	*31 (4.9)*	*26 (5.6)*			
Mental fatigue sub-score, *mean* ± *SD*	4.6 ± 1.7	3.9 ± 1.5	-0.6	-0.9, -0.4	˂0.01
*Missing*	*33 (6.4)*	*27 (6.0)*			
**Insomnia**					
Total score, *mean* ± *SD*	8.1 ± 6.1	4.4 ± 4.3	-3.8	-4.5, -3.1	˂0.01
No clinical insomnia	261 (54.8)	328 (78.3)			
Sub-threshold insomnia	137 (28.8)	82 (19.6)			
Moderate/severe clinical insomnia	78 (16.4)	9 (2.1)			
*Missing*	*41 (7.9)*	*33 (7.3)*			

Statistics: Independent sample T-test for continuous variables, Fishers Exact Test for categorical variables, Pearsons Chi-squared test for dichotomous variables, and Mann–Whitney U-test for ordinal variables

*Abbreviations*: *SD* Standard Deviation, *CI* Confidence Interval

The most frequently reported mental health issue in both groups was insomnia. Almost half of the CMP patients reported sleeping problems compared to one in four of the patients without CMP. A significantly higher amount of CMP patients (16.4%) than patients without CMP (2.1%) reported sleeping problems corresponding clinical insomnia (Table 2). Among CMP patients, 12.3% reported clinical anxiety, and 5.1% reported clinical depression, compared to 3.4% and 0.7%, respectively, among patients without CMP. Both physical and mental fatigue was significantly more prevalent among CMP patients, compared to patients with no CMP (Table 2).

### Associations between number of pain sites and pain intensity, and symptoms of anxiety, depression, fatigue, and insomnia among the patients with CMP

Among CMP patients, we found that patients with high number of pain sites reported significantly more symptoms of anxiety, depression, fatigue, and insomnia, compared to the patients with fewer pain sites (Table 3). This tendency was not seen in patients who reported nine to twelve pain sites. Patients with six to eight pain sites reported the highest levels of mental health symptoms.

**Table 3:** Self-reported symptoms of anxiety, depression, fatigue, and insomnia among CMP patients in primary health care (*N* = 517), grouped by number of pain sites. Symptom score is presented for as mean and Standard Deviation (SD), number of cases of anxiety and depression are presented in number of participants and percent (%)

**Mental health symptoms**	**Number of pain sites**				
	**0–2** ***n* = 193**	**3–5** ***n* = 204**	**6–8** ***n* = 70**	**9–12** ***n* = 19**	
	*Mean* *(SD)*	*Mean* *(SD)*	*Mean* *(SD)*	*Mean* *(SD)*	*P*-value
**Anxiety score**	3.9 ± 3.0	5.6 ± 4.0	8.3 ± 4.7	3.9 ± 3.7	˂0.01
Borderline anxiety^a^, n (%)	10 (5.1)	21 (10.6)	17 (23.6)	4 (20.0)	˂0.01
Clinical anxiety^b^, n (%)	0	3 (1.5)	5 (6.9)	0	˂0.01
**Depression score**	2.1 ± 2.4	3.4 ± 3.4	5.5 ± 4.0	4.4 ± 3.9	˂0.01
Borderline depression^a^, n (%)	3 (1.6)	10 (4.9)	9 (12.9)	2 (10.5)	˂0.01
Clinical depression^b^, n (%)	0	1 (0.5)	0	0	˂0.01
**Mental fatigue score**	3.9 ± 1.3	4.4 ± 1.5	5.8 ± 2.2	5.5 ± 2.7	˂0.01
**Physical fatigue score**	7.6 ± 3.0	10.0 ± 3.6	12.3 ± 3.6	11.3 ± 4.5	˂0.01
**Insomnia score**	4.8 ± 4.6	8.5 ± 6.1	11.6 ± 6.7	10.5 ± 5.3	˂0.01

Statistics: Ordinal variables were analysed a Kruskal–Wallis one way ANOVA, adjusted for the Bonferroni correction for multiple tests, Pearsons Chi-squared test was used for dichotomous variables. Missing: 31

^a^Borderline anxiety/depression; defined as Anxiety/Depression score between 8–10

^b^Clinical anxiety/depression; defined as Anxiety/Depression score ˃ 11

For the CMP patients, the higher pain intensity, the higher mental health symptom levels were reported (Table 4). A marked increase in symptom scores were also seen for depression and insomnia, when shifting from moderate to severe pain intensity.

**Table 4:** Self-reported symptoms of anxiety, depression, fatigue, and insomnia among CMP patients in primary health care (*N* = 517), grouped by pain intensity. Symptoms score is presented as mean and Standard Deviation (SD), number of cases of anxiety and depression are presented in number of participants and percent (%)

**Mental health symptoms**	**Pain intensity**				
	**None/very mild** ***n* = 222**	**Mild** ***n* = 185**	**Moderate** ***n* = 68**	**Severe** ***n* = 3**	
	*Mean (SD)*	*Mean (SD)*	*Mean (SD)*	*Mean (SD)*	*P*-value
**Anxiety score**	4.4 ± 3.4	6.1 ± 4.1	7.2 ± 4.4	7.7 ± 4.9	˂0.001
Borderline anxiety^a^ n (%)	11 (5.0)	25 (13.5)	14 (20.6)	1 (33.3)	˂0.001
Clinical anxiety^b^ n (%)	2 (0.9)	3 (1.6)	3 (4.4)	0	˂0.001
**Depression score**	2.5 ± 2.5	4.1 ± 3.8	4.2 ± 3.2	8.3 ± 4.2	˂0.001
Borderline depression^a^ n (%)	4 (1.8)	15 (8.0)	4 (6.0)	1 (33.3)	˂0.001
Clinical depression^b^ n (%)	0	1 (0.5)	0	0	˂0.001
**Mental fatigue score**	4.2 ± 1.3	4.7 ± 2.0	5.1 ± 2.0	6.0 ± 3.6	˂0.001
**Physical fatigue score**	8.5 ± 3.0	10.4 ± 3.9	11.7 ± 3.7	13.3 ± 2.1	˂0.001
**Insomnia score**	5.8 ± 4.7	9.3 ± 6.2	11.6 ± 6.5	17.7 ± 5.9	˂0.001

Ordinal variables were analysed by a Kruskal–Wallis one way ANOVA, adjusted for the Bonferroni correction for multiple tests, Pearsons Chi-squared test was used for dichotomous variables. Missing: 31

^a^Borderline anxiety/depression; defined as Anxiety/Depression score between 8–10

^b^Clinical anxiety/depression; defined as Anxiety/Depression score ˃ 11

## Discussion

In this cross-sectional study among patients of general practitioners in primary care, just over half reported CMP symptoms. The majority of the included study participants reported good mental health, with few cases indicating clinical disorders. However, patients with CMP reported significantly higher symptoms on all the studied mental health issues compared to patients without CMP. Symptoms of anxiety, depression, fatigue, and insomnia increased with increasing number of pain sites and pain intensity. Within the sub-groups of number of pain sites and pain intensity, a larger amount of patients reported symptoms representing risk of developing clinical anxiety, with increasing number of pain sites and pain intensity.

### Strength and limitations

Our major strength is our study population. We have assessed an unspecified group of CMP patients in general health practice with no exclusion criteria related to type, duration or severity of CMP to highlight the general symptomatic multifactorial burden this group is representing. In the current study we categorized patients with CMP complaints regardless of pain site in order to explore common mental health symptoms in these patients. Our results may therefore present a picture of mental symptoms for these patients overall. Although the results cannot be directly generalized to site-specific CMP complaints, it is well documented that prognostic factors are shared among the different complaints [25, 26]. Thus, the study may point to important features of mental health issues that need attention in CMP patients regardless of pain site. Other strengths of the present study are the use of validated and reliable questionnaires developed for CMP patients, and the comprehensive amount of data collected, describing pain- and mental health characteristics in detail. The presented data, adds to previously published findings from this study showing significantly lower health-related quality of life among CMP patients compared to patients without CMP [32].

There are some limitations in our study. Firstly, by using a cross-sectional design, we are not able to claim causality or certain relationship between CMP and symptoms of impaired mental well-being. Secondly, a high proportion of the GP’s contacted declined to participate in our study, and we have no information on their reason for non-participation. There is a possibility of these general practitioners not fully representing the general group of practitioners in Trondheim county. Perhaps did we include general practitioners with a high amount of patients with CMP or with a special interest and awareness of this patient population. Only 23% of the eligible patient population agreed to participate, which may introduce selection bias into our study, and thus reduce the generalizability of our findings. Thirdly, like most observational studies, the use of self-reported information may introduce some bias to the material. There are individual, social, and cultural differences in how CMP, pain, fatigue, insomnia, anxiety and depression is evaluated and perceived. Although the factors assessed in the current study, symptoms of anxiety, depression, fatigue and insomnia, are highly dependent on subjective perception, they are important prognostic factors and relevant for proper management of these patients in clinical practice.

Another important limitation is that the CMP patient group could have experienced CMP at different time points during the past year, however the questions regarding mental health referred to the last week or month. Therefore, patients with CMP several months ago may not report any reduced mental health symptoms in present time although they could have been present at the time of CMP. Another limitation is the heterogeneity of both the CMP group and the group without CMP. The patients in the CMP group consists of both patients with current CMP at the time of fulfilling the questionnaire, while others in this group may be quite free of symptoms after experiencing CMP several months ago. This aspect will clearly affect the CMP group reports of mental health symptoms and information recalling. Further, it is likely that subjects in the no-CMP group could have experienced CMP previously and may still experience both somatic and metal symptoms caused by this disorders when reporting to our study. Patients invited to the study were informed that the objective was to address CMP. It is therefore perceivable that we recruited a higher proportion of patients with CMP than patients without CMP among the GPs’ patients. No sample size calculation was performed for the current study.

### Comparison with other studies

We found in our study that the patients with CMP reported significantly more symptoms of anxiety and depression compared to the patients without CMP. These results are supported by a number of previous studies reporting a higher risk of developing anxiety and depression among patients with CMP [45–47]. In the present study, the prevalence of clinical anxiety (12%) was higher than clinical depression (5%), though symptoms of depression increased considerably more with increasing pain intensity. However, in a comparable study assessing mental health issues in primary health care, Matthew and colleagues [48] found a higher prevalence of depression than anxiety (3%) in their population. They used a more comprehensive questionnaire (Symptom Checklist 20, SCL-20) than ours, and this may have enabled a more detailed examination of depression explaining the discrepancies. Few of our patients reported symptoms corresponding to clinical anxiety and depression. However, we found that 20–30% of the patients with moderate to severe pain intensity reported symptoms corresponding to borderline anxiety. This is in line with Barnett et al. who found in a study among primary health that anxiety is more prevalent among patients with joint pain, and that sever pain was related to clinical anxiety [49]. About 20% of our patients with 6 pain sites or more reported symptoms corresponding to borderline anxiety, and 10% symptoms corresponding to borderline depression.

Fatigue has been associated with severe and prolonged health disorders [9], and the CMP patients in our study had significantly higher mean total fatigue score, including both higher physical and mental fatigue sub-scores, compared to patients without CMP. A previous study assessing the prevalence of fatigue in the general Norwegian population considered the average total score of 0- to 11.2 points to be writing the normal range [50]. This indicates that the patients without CMP in the present study are within the upper margins of normal range and thus representative of the general population, whereas the CMP patients reported a higher than normal mean fatigue score of 14.2 points. Furthermore, we found increased fatigue symptoms with increasing number of pain sites. Chronic fatigue is previously found to be more prevalent among patients with multiple site CMP, compared to patients with single site pain [11]. This is in concurrence with our findings as the patients with the highest number of pain sites reported most symptoms of fatigue. However, we found the most pronounced increases in symptoms of physical fatigue, while the increase in symptoms of mental fatigue were more modest. Fatigue seem to be a predominant factor in many severe health issues, especially if it is combined with symptoms of anxiety and depression [11], while vitality somehow seem to protect against several severe health conditions [9, 51].

Insomnia is common among patients with CMP. Insomnia has been identified as a contributor to chronic pain, and perceived pain intensity in particular [12–14, 22]. In our population, nearly half of the CMP patients reported sleeping problems, and 16% reported moderate to severe clinical insomnia. This is in concurrence with previous research showing a negative effect of pain on sleep quality [14, 46]. A cohort study by Asih and collegues [52], including 325 patients with CMP, revealed a high prevalence of moderate to severe insomnia among the participants, but the association with pain intensity was not clear. Our results showed that symptoms of insomnia increased with increasing pain intensity. The Norwegian HUNT study including 8563 women and 7598 men, revealed a strong association between CMP and insomnia. This association was most pronounced in the working population with a high prevalence of work-related fatigue [53]. The mean insomnia symptom score was higher when categorized according to pain intensity rather than number of pain sites.

The mental health and pain-related outcomes included in our study share common risk factors. Insomnia and symptoms of depression are found to increase the risk for developing CMP [14], and to increase perceptions of pain and daily life disabilities [52]. Causal relationships between sleep quality and pain has been documented in both directions [54]. Moreover, tendency of somatization and adverse health beliefs, are found to be determinants for unspecified CMP [55]. On the other hand, anxiety and depression are found to be independent predictors of reduced quality of life among CMP patients [23]. Several studies conclude that CMP leads to limitations in daily life activities which in turn may affect the patients’ both physical and mental state of health, and the ability to cope with and recover from chronic pain [29, 30]. Our study in a unspecified group of CMP patients in the primary health care system confirm the major burden of CMP related to mental health found in studies in more pre-specified patient groups.

### Interpretation

Our findings show that patients in general practice with CMP are considerably more burdened by different mental health related issues than patients without CMP. Increased awareness of the complexity of CMP and its relation to impaired mental health is important for primary health care personnel in order to provide appropriate patient management. The observed increase in levels of mental health symptoms with increasing pain intensity and number of pain sites highlight the importance of the primary health care system establishing effective strategies for managing mental health issues among patients with CMP, and particularly for patients with high pain symptom levels.

### Generalisability

Our study aimed to include a general group of primary health care patients, representing the working population, and included few inclusion criteria. Although, it is possible that a rather high number of patients who volunteered for participation compared to the general population have experienced musculoskeletal pain, due to this being a study on chronic musculoskeletal pain. However, patient demographic characteristics of our study indicate that especially the present CMP population to be representative for the general Norwegian working population’s use of the primary health care system due to musculoskeletal complaints [29, 46, 48, 56]. We believe our findings may be generalizable to the general working population with CMP seeking care in the primary health care system.

## Conclusion

Among patients in primary health care general practice, we found that patients with CMP reported more symptoms of anxiety, depression, fatigue and insomnia compared to patients without CMP. Higher number of pain sites and increased pain intensity were related to more symptoms of impaired mental health, especially insomnia and borderline anxiety. In terms of impaired mental health, pain intensity differentiated the pain subgroups more than number of pain sites. Our findings underscore that the burden of CMP also involves multiple aspects of mental health, implying that health care personnel need to pay attention to mental health issues in the management of CMP patients. Clinical management of this patient group should not be limited to somatic pain and limitations in physical function, but also raise awareness for potential mental health issues affecting the rehabilitation process.

## Data Availability

The data that support the findings of this study are available from Siv Mørkved, but restrictions apply to the availability of these data, which were used under license for the current study, and so are not publicly available. Data are however available from the authors upon reasonable request and with permission of Siv Mørkved.

## Abbreviations

CMP: Chronic musculoskeletal pain
GP: General practitioners
